# The use of regional platforms for managing electronic health records for the production of regional public health indicators in France

**DOI:** 10.1186/1472-6947-12-28

**Published:** 2012-04-03

**Authors:** Marie-Hélène Metzger, Thierry Durand, Stéphane Lallich, Roger Salamon, Philippe Castets

**Affiliations:** 1 Université Lyon I - CNRS-UMR 5558, Laboratoire de Biométrie et Biologie Evolutive, Lyon, France; 2 Direction du Système d'Information, Centre Léon Bérard, Plate-forme SISRA, Lyon, France; 3 Université Lyon 2 - Laboratoire ERIC, Lyon, France; 4 ISPED-Centre INSERM U 897 - Université Victor Segalen Bordeaux 2, Bordeaux, France; 5 Haut Conseil de la Santé Publique, Paris, France; 6 Direction du Système d'Information, Hospices Civils de Lyon, Plate-forme SISRA, Lyon, France

## Abstract

**Background:**

In France, recent developments in healthcare system organization have aimed at strengthening decision-making and action in public health at the regional level. Firstly, the 2004 Public Health Act, by setting 100 national and regional public health targets, introduced an evaluative approach to public health programs at the national and regional levels. Meanwhile, the implementation of regional platforms for managing electronic health records (EHRs) has also been under assessment to coordinate the deployment of this important instrument of care within each geographic area. In this context, the development and implementation of a regional approach to epidemiological data extracted from EHRs are an opportunity that must be seized as soon as possible. Our article addresses certain design and organizational aspects so that the technical requirements for such use are integrated into regional platforms in France. The article will base itself on organization of the Rhône-Alpes regional health platform.

**Discussion:**

Different tools being deployed in France allow us to consider the potential of these regional platforms for epidemiology and public health (implementation of a national health identification number and a national information system interoperability framework). The deployment of the Rhône-Alpes regional health platform began in the 2000s in France. By August 2011, 2.6 million patients were identified in this platform. A new development step is emerging because regional decision-makers need to measure healthcare efficiency. To pool heterogeneous information contained in various independent databases, the format, norm and content of the metadata have been defined. Two types of databases will be created according to the nature of the data processed, one for extracting structured data, and the second for extracting non-structured and de-identified free-text documents.

**Summary:**

Regional platforms for managing EHRs could constitute an important data source for epidemiological surveillance in the context of epidemic alerts, but also in monitoring a number of indicators of infectious and chronic diseases for which no data are yet available in France.

## Background

France's *Public Health Act *of August 9, 2004 [1] introduced 100 health targets for the 5-year period from 2004 to 2008. This law is under evaluation, and new public health targets should be established shortly. The law has given impetus to an evaluation process of public health programs in France at the national and regional levels. Specific indicators for measuring and monitoring the results have been defined. The health problems that are the focus of these 100 targets are very diverse, covering the population's entire range of health conditions. Some targets deal with health determinants (alcohol, tobacco, physical activity, nutrition, etc.), infectious diseases, maternal and child health, cancer pathologies, cardiovascular diseases, etc. Certain targets also focus on evaluating the healthcare system itself (iatrogeny, antibiotic resistance, pain management).

This national approach was accompanied by the implementation of regional public health plans. Each region was required to define priority areas, taking into account the health status of the region's population. However, the regions were confronted with the reality that few regional indicators were in fact available. In 1 report, the French High Counsel for Public Health points out that the difficulties in quantifying regional targets are not linked to a lack of available data but rather to the underuse of these data [2]. The reasons are the same as those described in the United States by Friedman and Parrish [3]: "population health data are scattered widely at various agencies and web sites, in various forms, at various geographical levels, and with various statistical and reporting conventions". Of the 100 quantified targets defined in the 2004 law, 28 were submitted to a preliminary production of epidemiological or scientific information not available in 2009.

The introduction of regional platforms for managing electronic health records (RPF-EHRs) constitutes a new health information system, of which this article proposes to address certain design and organizational aspects, permitting us to consider their potential in regional epidemiological development. The article will base itself on organization of the Rhône-Alpes regional health platform (SISRA) to demonstrate its feasibility.

## Discussion

In this article, an electronic health record (EHR) is defined [4] as "a repository of information regarding the heath status of a subject of care, in computer-processable form".

**Regional platforms for managing electronic health records **(RPF-EHRs) represent a tool that lets us: [*translation*] "group together all applications and environments that can be used by the same community of health-professional users and institutions as a means of sharing and integration" [5]. The RPF is a tool that converts non-shareable EHRs to shareable EHRs at level 3 of the International Organization for Standardization (ISO) definition for EHRs (across different EHR locations and/or different EHR systems) [4].

The interoperability of health information system is defined as: [*translation*] "the capability of heterogeneous systems to interchange data in a way that the data from one can be recognized, interpreted, used and processed by other systems." EHRs are created from very heterogeneous data within a healthcare structure, whether in medical offices or healthcare institutions and even more so from one facility to another, combining structured and non-structured data in various electronic formats. To ensure interoperability between these systems, data-sharing norms and standards must be respected to identify a certain amount of information regarding the context in which the medical record was filled out [6].

Transmitting the following complementary information is essential to the correct interpretation of patient data: type of document (surgery report, consultation letter, imaging report), its creation date and location (institution, department), the health professional who generated the information and the chaining of documents relative to one another. EHR designers must therefore be able to associate the internal architecture of specific EHRs of the system they are proposing with an external architectural standard that will allow standardized data transfer. In France, the shared health information systems agency ASIP Santé has defined an information system interoperability framework [7] as a set of specifications regarding content (semantics, syntax and format of shared contents), types of interoperable services (records management, access clearance to EHRs, document-sharing and interchange), data transfer (interconnection protocols, synchronous or asynchronous data routing). This framework is expanding progressively. In terms of content, the standard selected is Clinical Document Architecture Release 2 (CDA R2) with Extensible Markup Language (XML) syntax for clinical documents (including an inseparable heading and body). In terms of "service" interoperability, the following metadata are associated with shared or interchanged electronic documents: national health identification number (INS), actions relating to the documents, the practical framework for carrying out these actions, disease diagnosis, type of document and author's profession/specialty. These metadata also permit control of access rights to documents. The interoperability profile selected for medical document interchange or sharing is the Cross-enterprise Document Sharing (XDS) Integration Profile defined by the IHE (The Integrating the Healthcare Enterprise). The XDS profile is neutral to document content or format (structured data, text or image), allowing all types of medical information to be transferred.

Deployment of RPF-EHRs began in the 2000s in France. Some needs related to the set-up of various national plans were identified, notably as a consequence of health crises (2004 emergency plans after the summer heat wave) or as part of certain public health programs, especially the 2003-2007 oncology plan (implementation of shared cancer-related medical information) [5].

The regional Rhône-Alpes RPF-EHR was developed in this context. The target was to share EHRs across different hospitals and with ambulatory care practitioners.

A new element gave fresh orientation to the RPF-EHR concept. It was the law of March 4, 2002, regarding patients' rights to consult their medical records. Following this law, the "personal medical record" (DMP) concept was introduced with the law of August 13, 2004. It corresponds to the ISO definition of integrated care EHRs. However, some conditions were attached to the concept. The DMP is the property of the patient and not of the health professional, that is, it is the patient who decides to create it and authorizes health professionals to enrich it with documents that they consider relevant to the coordination of care. The patient can mask some information in his/her DMP if he/she judges that it could be disadvantageous to his/her relationship with the health professional. The concept mixed 2 different aspects of EHRs: health information exchange between health professionals, labelled as EHRs in the text below, and consumer health records entered by the patient himself/herself, termed personal health records in the text below. Confusion between these 2 types of functionalities is a major reason for difficulty in deploying this tool at the national level in France.

The law of August 13, 2004, in the context of regionalizing health policies, was the next step in promoting the development of RPF-EHRs. The Rhône-Alpes region developed RPF-EHRs but without mixing the 2 aspects of the DMP. The system is based on the principle of an EHR for sharing medical information between health professionals. According to the law of March 4, 2002, patients can consult their EHR but cannot manage it themselves.

### Regional medical records management platform in Rhône-Alpes: SISRA

The objective of this section is to describe the organizational aspects of SISRA.

#### Key element: mode of governance

In the French Rhône-Alpes region, regional projects were managed until 2005 by the regional hospitalization agency (ARH) and the regional union of health insurance funds (URCAM), under no explicit architecture or information system master plan. In other words, initiatives could overlap or even be duplicated at times. When the fourth regional health master plan was developed, the ARH mandated a team of doctors and technicians to build a health information system master plan for the region. Governance was officially established on March 5, 2005, combining the ARH, the URCAM, the Rhône-Alpes region and the regional union of liberal doctors (URML), as well as the Regional Committee of Users (CISS-RA), into 1 steering committee. Governance falls under the supervision of the GCS (Groupement de coopération sanitaire) SISRA (Système d'Information de Santé en Rhône-Alpes). This health cooperation group is made up of representatives from university hospitals and general medical practice. Every member of the GCS SISRA heads technical development projects in collaboration with industrialists. Each month, the steering committee and GCS SISRA evaluate the evolution, implementation and deployment level of the region's projects.

The SISRA platform was built around a few intangible principles, established by the steering committee, among which the following should be retained:

- Pooling of means and tools: as much as possible, tools should be reusable. All Rhône-Alpes networks thus rely on the same tool (PEPS: external data storage software) adapted to the operational needs of network promoters.

- Non-interference in the institution's policy on computerization of information systems. However, the governing body is pushing for computerization of non-equipped facilities, interfacing of their information systems with the platform and progressive data entry into the platform. All facilities are encouraged to become equipped with EHRs. The smaller facilities can choose from two methods: either an invitation to tender to select a common supplier or the PEPS module, which hosts simpler EHRs.

**Figure 1:**
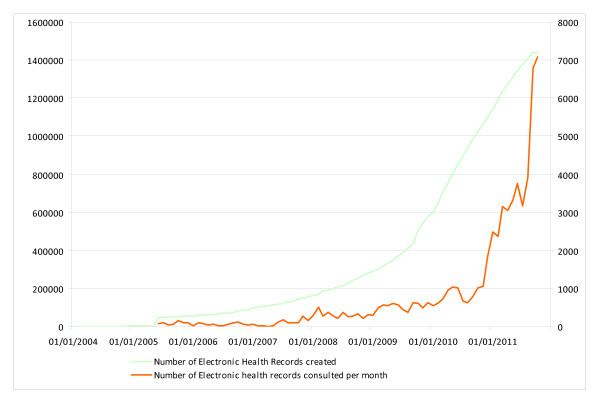
**Number of EHR created and number of consultations per month in the SISRA platform from 2004 to 2011**.

Figure 1 illustrates this organization's efficiency. The curve begins with the device going through a 3-year latency period (corresponding to the classical credibility threshold) and then taking off exponentially. In August 2011, 111 sites were feeding regional records, and 2.6 million patients were uniquely identified (42.6% of the regional population), including 1.3 million patients with medical content. The curve of medical records consultation follows the same trend as the creation of EHRs but with a certain lag due to the fact that a critical volume of medical records is necessary to convince practitioners to use the tool in their practice. To increase this volume, the governing body decided that all emergency room visit reports, interdisciplinary oncology meetings (RCPs) and childbirth reports should be switched over to the platform in 2008/2009. In less than 18 months, the decision led to RCP reports being shared regionally almost exhaustively (from less than 30% of the region's 44 institutions organizing RCPs to 90% between May 2009 and December 2010).

#### Rationale for SISRA's organizational and technological choices

SISRA's technological and organizational choices are partly the result of an inventory carried out in Rhône-Alpes in 2004. A survey of the region's 300 health institutions indicated that 58% of them were level 0 to 2 (data sharing not possible), and 42%, level 3 to 5 (communicating medical information) [8]. Faced with this heterogeneity, GCS SISRA opted to implement an extremely flexible system, allowing all institutions, regardless of their computerization level, to be integrated into RPF-EHRs.

#### Technical description of the SISRA medical records management platform

The aim of this regional platform is to allow medical practitioners to share EHRs with other medical practitioners (other public hospitals or private clinics, primary care practices) who need medical information to coordinate patient care.

**Figure 2:**
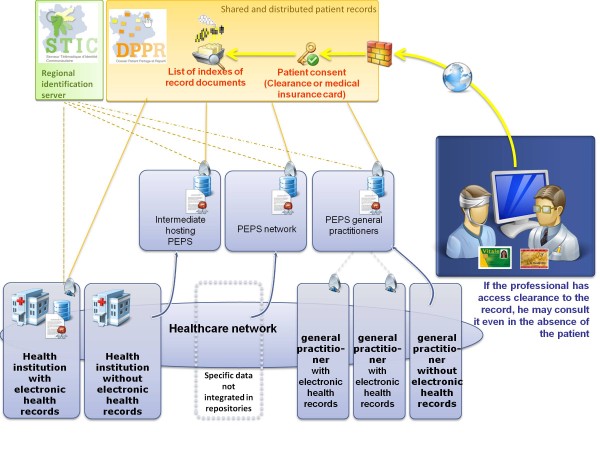
**Organization of the regional SISRA platform**. PEPS: external data storage software.

The fundamental RPF-EHR principle is to not interfere with the hospital information system (HIS) in healthcare facilities. The platform is fully described in another publication [9], and we will briefly mention its essential aspects here (Figure 2). For level 3 to 5 health institutions, a direct connection was established between the HIS and the platform with connectors developed in partnership with the software editors of each corresponding institution. For level 0 to 2 institutions, PEPS, also linked to the platform by connector, was introduced [9]. These connectors were created by more than 20 medical record editors funded by SISRA in the amount of €500,000. To simplify the creation of connectors, a device called a "gateway" was integrated between medical records software and the regional platform. This device accepts the input of standardized formats, handles processes specific to the region, such as connection to STIC, and controls communications with regional and national devices. It helped reduce the price of the connectors from €100,000 for the first ones to €15,000. This connecting device linked to a gateway does more than simply feed regional and national records - it also enables the site to retrieve data from outside. Bidirectional connection is established between the institution and its external environment.

The platform includes other tools as well: the regional health identification number module (STIC) associated with an identification chart, available in all the region's input offices; shared and distributed medical records (DPPRs), which store only metadata and facilitate browsing on all local repositories; and the PEPS module, which allows healthcare networks and general medical practices to access a common tool perfectly tailored to their specific needs. Other projects have piggybacked on this momentum. Particularly noteworthy is the *Trajectoire *project, a veritable "marketplace" that facilitates referral of short-term patients downstream to follow-up- and rehabilitation-style facilities. To date, 17 French regions from 21 French metropolitan areas have opted for the tool.

### Design and development of a regional epidemiological platform

The objective of this section is to describe the organizational aspects of implementing a regional epidemiological platform (RPF-EPI) based on RPF-EHRs of the Rhône-Alpes region.

As part of a regional health agency pre-configuration framework, the use of regional data for public health purposes is under consideration. The regional health surveillance and alert server (known as OURAL) could thus analyze data and check the balance between bed supply and demand by health area. That said, the monitoring station created on top of the *Trajectoire *tool is beginning to show certain weaknesses in terms of care in the region: available bed capacity in a rehabilitation-style facility does not automatically mean that any need can be filled. Finally, the need to measure healthcare efficiency in certain areas (e.g. treatment of strokes, chronic diseases, etc.) is becoming clear and, naturally, regional decision-makers are turning to GCS SISRA to study the feasibility of setting up a platform for epidemiological processing in the next regional master plan. In fact, in Rhône-Alpes, 12 chronic diseases represent 40% of the region's healthcare spending and affect 10% of its population, i.e. 600,000 patients. The purpose of this epidemiological platform is to build a new information system which corresponds to the ISO definition of a population health record (popHR) [3,4]: "a popHR contains aggregated and usually de-identified data. It may be obtained directly from EHRs or created de novo from other electronic repositories. It is used for public health and other epidemiological purposes, research, health statistics, policy development, and health services management."

**Figure 3:**
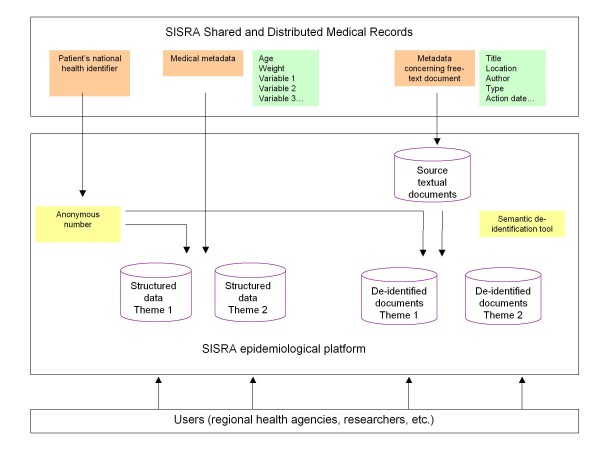
**Organization of the SISRA epidemiological platform**.

We will address the various steps required to transfer data from RPF-EHR to the RPF-EPI (Figure 3), which will permit popHR to be built.

#### Governance of the regional epidemiological platform

Given the considerable volume of data to process and the servers' current data-processing capabilities, the regional level appears well-adapted for setting up epidemiological platforms. This geographical level is also coherent with regional strengthening of public health policies, allowing flexible organization of regional databases according to public health priorities established in the regional public health plans. GCS SISRA has the infrastructure needed to manage system operations (data security, backups, metadata documentation, etc.) and can therefore perform this function. Policy governance is also necessary, and a steering committee consisting notably of regional public health decision-makers will be formed.

#### Extracting data from SISRA-shared and distributed medical records

Data will be extracted from SISRA platform DPPRs. As soon as the DPPR is open, patients are advised that their de-identified data may be used for epidemiological purposes and are given the option to refuse.

To allow pooling of heterogeneous information contained in various independent, local repositories, it was necessary to define the format, norm and content of the metadata to be transmitted to the DPPR. Defining the metadata meant finding a balance between detailed data requiring a highly-structured information system source and rough data available in all data repositories but not permitting high-performance data processing. The solution adopted was to always retrieve the most information possible while accepting that not all systems would necessarily provide the same level of detail. This can be demonstrated through the example of imaging reports, where some systems have detailed information, such as modalities (scanner, magnetic resonance imaging, lung x-ray, etc.) or the body part examined. The minimum mandatory metadata selected were "imaging exams," proposing that more advanced sites add complementary information in the form of comments. These comments are widely used when browsing through medical records to choose which exam to view.

XML technology permits us to define variable information content, to specify where each data repository must provide the minimum, mandatory information, but can also be completed with available complementary data. For example, the metadata related to type of documents allows the distribution of each type of document available in the platform to be described: 19.1% (consultation reports), 9.5% (hospital discharge reports), 7.7% (imaging reports), 7.5% (emergency department visit reports), etc. This device also permits a data repository to evolve over time with complementary information as soon as it is able to produce it without the interface being re-engineered or central teams being called in. It is therefore an operational device that retrieves the most detailed information available in 1 model, regardless of the choice of information systems made by a large number of sites in the region.

Given the architecture of the SISRA platform, data can be selected only by using the metadata stored in the DPPR, allowing data filtering and chaining. Regional EHRs at the patient level are virtual EHRs in the sense that regional DPPRs store only metadata and facilitate browsing on all local repositories. Conversely, the epidemiological platform (popHR) will generate a single database related to the public health topic studied. Because of the regional health identification number generated by STIC, it is possible to follow each patient over time and to build cohort studies. The patient's EHR selection process for popHR will be carried out at the SISRA platform level and will be followed by a process of data de-identification. This process should be under the responsibility of GCS SISRA.

#### Data de-identification

In epidemiological studies, medical data-chaining and identification of duplicates are essential. These 2 points rely on a unique patient identification number. The need for a national health identification number has been the object of widespread mobilization of French epidemiologists in the last 10 years, notably when DMPs were created [10]. Various methods have been proposed [11]. In its conclusions of February 20, 2007, the Commission Nationale de l'Informatique et des Libertés (CNIL) excluded the national social security number as the national health identification number and advocated the creation of a specific INS to be generated from the national social security number. Article L1111-8-1 of the public health code defines the regulatory framework for the creation of the INS: [*translation*] "An identification number of health-insurance beneficiaries who are under the care of a health professional or medical institution or are part of a health network is used for storing, hosting and transmitting health information. It is also used to open and maintain personal medical and pharmaceutical records." From this regulatory framework, ASIP Santé recently defined the INS development program [12]. An individual INS will be assigned to each health insurance beneficiary for life, and will be non-identifying, that is to say, it will be impossible to deduce any information from this identification number, and knowledge of an INS will not enable anyone to match it to the social security number. This identification number will be randomly generated (INS-A) from a nationally-centralized system for each health insurance beneficiary independently of his/her encounter with the healthcare system. The timetable for implementation of this INS is the responsibility of ASIP Santé and is not yet established to date. In the meantime, to continue rolling out health information systems, a temporary INS has been implemented. This temporary INS, so-called "calculated" INS (INS-C) was introduced in 2010 and is generated by an algorithm taking into account the patient's social security number, first name and birth date. The INS-C is generated at the first resort to care (outpatient or inpatient). The DMP is then actually generated on patient encounter with the health care system using the INS-C. When the INS-A becomes available, it will be theoretically possible for patients to create their own DMP without encountering the healthcare system but this mode of creation is not defined for now. In Rhône-Alpes, pending the availability of an INS, STIC was created. It assigns a regional unique health identification number. There are no plans for patients to create a regional EHR without encountering the healthcare system in Rhône-Alpes.

The CNIL wrote a note regarding the current status of personal data de-identification procedures in the public health sector, which is posted on its website under: *l'état des lieux en matière de procédés d'anonymisation*. The note discusses recommended de-identification procedures, notably those involving a secret-key hashing function. This operation consists of calculating a numerical value (a number) from a person's direct or indirect personal information (family name, given name, birth date, etc.), with the value then substituting the information from which it was calculated.

In some epidemiological studies requiring analysis of free-text data in the report (notably in research aiming to automatically extract epidemiological data [13,14]), it is essential to consider a more sophisticated de-identification procedure within the report. In the United States, confidentiality and security regulations regarding medical information are governed by the *Health Insurance Portability and Accountability Act *[15]. This law defines the following 18 types of identifiers as protected health information that must be deleted for the data to be used in research: family name and given name, geographic subdivisions smaller than a State, dates (excepted year) directly related to patient, age if over 90, telephone and fax numbers, electronic mailing addresses, social security number, medical record number, health plan beneficiary number, account number, certificate/license numbers, vehicle identifiers and serial numbers, device identifiers and serial numbers, Web URLs, Internet Protocol address numbers, biometric identifiers including finger and voice prints, full face photographic images and any comparable images, any other unique identifying number, characteristic, or code, except as permitted under HIPAA to re-identify data (specific scars or tattoos). In France, legislation has not precisely defined which type of data should be removed to de-identify health information. The CNIL assesses the de-identification level required for specific research projects on a case-by-case basis. When developing a RPF-EPI, it is essential to include tools that automatically de-identify reports that will be analyzed by researchers. Various research projects aim to develop these tools [16], notably through text-mining techniques [17].

#### Building databases according to public health themes

Based on the same philosophy as that applied in developing the SISRA platform, GCS SISRA wants progressive and flexible implementation of the device that will allow for adjustments as EHRs and the SISRA platform continue to evolve. Employing RPF-EHRs for epidemiological purposes will certainly be a driving force in defining evolutionary needs at source and notably in adding metadata. Given this permanent evolution and the delays expected between the time when decisions are made and when they are actually implemented, designing a regional health warehouse does not seem appropriate in the current context. It was thus decided that databases would be set up and developed by epidemiological or public health projects. Two types of databases can be created according to the nature of the data used: databases for extracting structured data (metadata retrieval) and non-structured and de-identified free-text documents.

Medical records are an obvious source of considerable information for collecting data on risk factors, symptoms, diagnoses and therapeutic patient care [18]. Extraction of relevant data for epidemiological or public health development is nevertheless greatly limited by the absence of structure in medical records [19]. Employing free-texts to describe patient follow-up is the most common practice. To enable statistical processing of data from these reports, medical information in free-text documents must be standardized. Given current developments in the automatic processing of natural language, these tools could potentially be exploited to extract data for epidemiological purposes [14,20-24]. Free-text data retrieval in the medical sector is the subject of many projects. However, there are various levels of sophistication in the methods deployed. For example, MedLEE (Medical Language Extraction and Encoding System), a system designed for processing free-text medical data, extracts information from unstructured text reports. Extraction depends on designated entities being recognized, but with no relationships established between the extracted entities [25]. Other experiments rely on semantic analysis methods [14,26,27]. Approaches are diverse, but are based on a common method generally used in automatic processing of free-text data. More specifically, linguistic analyses generally consist of the following processes:

- Segmentation of free-text data into lexical units (single words, compound words).

- Assignment of a unique label to these lexical units (morphological analysis and part-of-speech tagging).

- Syntactic analysis, on the one hand, allowing lexical units to be organized into syntactic domains and, on the other hand, linking these various syntactic domains according to their grammatical relationships.

- Semantic analysis that relies on previously-calculated linguistic information and permits the abstraction of extracted syntactic relationships into more general relationships (thematic roles, interactions between entities, etc.).

A few experiments have been conducted regarding the development of information-extraction systems based on semantic analysis methods for English-language texts. Cohen et al. [14] have reported very effective results with this type of tool, with an F-Score between 0.97 and 1.0 for various categories (anatomical site, histology, dimension, primary tumour, etc.) of anatomic pathology reporting in cancer, automatically extracted from a MedTAS/P (Medical Text Analysis System/pathology), unstructured text search engine based on Unstructured Information Management Architecture technology developed by IBM. Once the texts are analyzed with a view to extracting relevant elements and the relationships between them, the next step consists of retrieving these data to populate a knowledge base, such as a relational database [14]. For the French language, the Xerox Incremental Parser tool, developed by Xerox and currently being adapted to the medical field as part of a research project funded by the Agence Nationale de Recherche (ALADIN-DTH project), is based on the same principle [20].

These natural language processing tools must rely on standard terminologies to ensure data standardization and allow statistical processing. Many French language health terminologies are available [28], each built for a specific application, which limits their utility for other purposes. Multi-terminology servers are being developed and should allow them to be employed to standardize the medical information contained in free-text documents with sufficient accuracy. One experiment on detecting hospital-acquired infections is under way as part of the ALADIN-DTH project that we are currently conducting [21], and preliminary results are demonstrating its feasibility [29].

#### Adapting epidemiological analysis techniques to the characteristics of EHR-generated data

Data extracted from EHRs are often from various sources and heterogeneous in nature (numbers and texts, but also images and sometimes sound). They are large in size and in number of cases, due to case accumulation and automatic, partial acquisition of the data. Furthermore, the data are often temporal, or even spatio-temporal. These characteristics require tailored solutions. Extracting knowledge from databases (Knowledge Data Discovery) is an approach that gives us a glimpse of new medical data-processing methods in the field of epidemiology [30-32]. First, data organization and storage must allow for the semi-structured nature of part of the data, by adopting XML language, for example, and must facilitate mining [33,34]. Several differences should be noted between mined data and data normally analyzed in statistics. Given their volume as well as their fragmented and often mechanical acquisition, the data contain atypical items (outliers), which impede generalization as well as many redundant or irrelevant variables. Data preparation is, therefore, an important step in the data-mining process, whether for locating and processing outliers [35] or for selecting relevant variables (feature selection) [36]. Whereas traditional epidemiological techniques factor in a small number of well-defined covariates, the inherent difficulty in extracting data from structured and unstructured text material lies in the diversity of covariates encountered and tested (more than 10,000, for example, in the study by Pakhomov et al. based on natural language processing to identify heart failure) [37]. Variable-selection techniques and data-mining methods, such as rules of association, the naive Bayesian model and neural networks [38], thus enable processing of a large number of covariates. Another particularity of the data is the large number of missing values, deriving from either improper filling, hospital structure, or missing values as such. For example, missing values could be processed by discretizing continuous variables and creating a missing values category. Generally, calculating p-values is not relevant, inasmuch as the data do not provide all the features of a true sample and a very large number of observations make any difference significant, which results in a multitude of p-values stuck at 0. Models are validated with separate datasets for learning and testing. If there are not enough data, cross-validation is an option [39]. Statistical tests to select discriminate variables also present the problem of controlling multiple risks for which many solutions have been developed in biostatistics [40].

In the medical field, datasets are most often imbalanced in the sense that the prior class probabilities are highly unequal [41]. In such cases, the performances of data-mining algorithms are lowered, especially the error rate corresponding to the minority class. For 2 classes, the minority class corresponds to positive cases, and the cost of misclassifying positive subjects is higher than the cost of misclassifying negative subjects. Solutions to class imbalance problems were proposed at both the data and algorithmic levels [42]. At the data level, these solutions change class distribution. They include different forms of re-sampling, such as over-sampling or under-sampling in a random or directed way [43]. At the algorithmic level, a first solution is to re-balance the error rate by weighting each type of error with the corresponding cost [44] In the case of decision tree learning, other algorithmic solutions consist of modifying the splitting criterion [45], adjusting probabilistic estimates at the tree leaf or adjusting decision thresholds [46]. In addition, it is necessary to employ more appropriate evaluation metrics, such as Receiver Operating Characteristic (ROC) curves, Area Under the ROC curve (AUC), Precision, Recall and F-measures [47].

For example, in the ALADIN-DTH project [20], 1 objective is to build a classifier which will allow distinction between infected and non-infected cases automatically through medical documents available on the platform. For this purpose, the method used in the project was to select 100 infected patients in 4 medical specialities (orthopedics, digestive surgery, neurosurgery, intensive care). The infected cases are validated on a regular basis by infection control practitioners with standardized national protocols of nosocomial infection surveillance [48,49]. Conversely, "non-infected" status is not systematically validated for other patients after their hospitalization. This fact is due to class imbalance. For example, for 100 patients with orthopedic surgical infections, it would be theoretically necessary to validate 24,900 non-infected patients (incidence rate: 0.4% in the region) [50]. In terms of workload and costs, it is not feasible. The method chosen for taking this point into account was to build the learning set by exhaustively selecting all infected patients and by random sub-sampling of non-infected patients. So we randomly selected 100 non-infected patients in each of the 4 medical specialties.

To build the classifier, we chose rules-based methods, particularly class association rules or decision trees. To elaborate these rules, we had to take dataset specificities into account with the different methods described above. This work is currently in progress. The validation of positive cases without validation of negative cases is a classical situation in such population surveillance systems. The experimental method for tracking nosocomial infections should be reproducible for other diseases by elaborating detection rules.

#### Current status of epidemiological platform use for public health in Rhône-Alpes

The regional architecture proposed is already used for metadata extraction, showing its feasibility. For example, analysis of RCP quality is ongoing with metadata related to document type. In fact, the RCP database has highlighted that at least 2 RCP reports were archived in the EHRs of 68% of patients who attended RCPs between March 2010 and February 2011. In accordance with French guidelines, only 1 RCP should be held at the time of cancer diagnosis for a multidisciplinary decision on the therapeutic management of patients. These deviations from the guidelines will be studied precisely in coming months to understand the reasons and possibly adjust the recommendations to clinical situations not envisaged in the current recommendations.

At present, the number of metadata available for epidemiological studies is limited in the platform for collecting public health indicators of the Public Health Act. The Steering Committee of the Epidemiological Platform is currently working to select relevant public health regional indicators from the 100 national health targets for which extraction from the platform could be considered. Building new metadata required for the extraction of selected indicators will be evaluated, and the feasibility of exploiting unstructured data studied. A shortlist of indicators from the Public Health Act, for which production from the regional platform would be relevant, has been drawn up (Table 1). This pre-selection takes into account both regional priorities in public health and specificities of the method for collecting these indicators with a regional platform for managing patient records. Indeed, specificity of the method is attributed to the fact that the platform covers mainly people with healthcare utilization. The platform does not yet cover the general population of Rhône-Alpes. Because of this selection bias, the Regional Epidemiological Platform does not yet answer the ISO definition of a popHR for the general population. As detailed by Friedman and Parrish [3], "population-based data on the social determinants of health needed for improving policy-making, program design, clinical care and health professional education" are not yet available.

**Table 1:** Indicators from 100 health targets under consideration for measurement with the regional platform for managing EHR

National French health target	Public health Indicator	Possible regional public health interventions
Target 5: Obesity: 20% reduction in the prevalence of overweight and obesity (body mass index (BMI) > 25 kg/m^2^) in adults: from 42% in 2003 to 33% in 2008	BMI of adults (≥ 18 years).	- Targeted medical management of overweight patients

Target 33: Reducing inequalities in illness and death by increasing life expectancy in groups faced with precarious situations	Probability of death and life expectancy by occupational categories, employment status, place of birth	- Prerequisite: collecting social information in EHRs - Prevention in targeted populations with precarious situation - Evaluation of actions implemented

Target 42: Vaccine-preventable diseases covered by immunization recommendations in the general population: achieve or maintain immunization coverage of at least 95% of appropriate ages in 2008 (i.e. 83 to 98%)	Immunization coverage rates in the general population and in the main risk groups	- Incentives to physicians for improved patient coverage - Information, awareness of target populations - Evaluation of actions implemented

Target 49: All malignant tumours: contribute to the improved survival of patients with tumours, including providing support for multidisciplinary oncology meetings of 100% of patients with a diagnosis of cancer.	- Rate of incident patients managed in multidisciplinary oncology meetings -Average survival rate at 5 and 10 years by type of cancer	- Targeted actions to improve cancer management - Evaluation of actions implemented

Target 72: Stroke: reduce the frequency and severity of functional impairment associated with stroke	Incidence and case fatality of stroke. - Frequency and severity of functional impairment and associated disabilities in the aftermath of stroke.	- Evaluation of stroke management - Planning regional needs of healthcare workers for managing patients based on their disability level - Evaluation of actions implemented

Target 74: Asthma: 20% reduction in the frequency of asthma attacks requiring hospitalization by 2008 (currently 63,000 full or partial hospitalizations per year)	Incidence of asthma attacks requiring full or partial (day or week) hospitalization	- Evaluation of actions implemented

Target 81: Reducing the impact of chronic kidney failure on quality of life of people, especially those on dialysis	Measuring quality of life of patients with chronic kidney failure and identifying the social problems associated with it	- Evaluation of actions implemented

**Figure 4:**
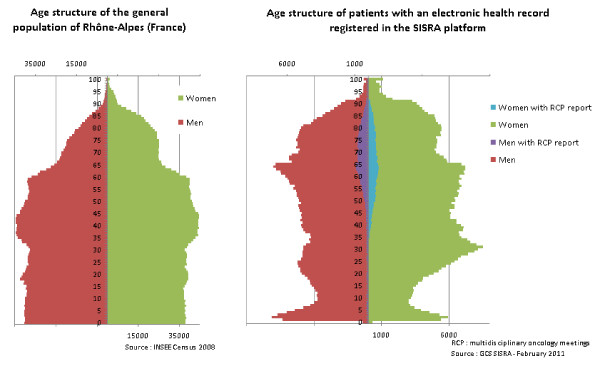
**Comparison of age structure of the general population and patients with an EHR registered in the SISRA platform in 2011**.

Figure 4 compares the age structure of the general population and the age structure of patients with an EHR registered in the SISRA platform in 2011. Globally, women are more representative of the general population than men. Per age group, old patients are well represented in both genders. The SISRA platform age structure essentially reflects healthcare utilization, which is the mandatory location for creating EHRs. This limit is important in the selection of indicators to be developed with the tool [3]. The design of a collection system of indicators, which monitor functional assessment and quality of life for patients with chronic diseases (malignant tumours, stroke, asthma), is thus currently most relevant.

However, this limit can be reduced if risk factors or prevention interventions in the general population (followed by the detection of cancer, cancer genetics consultations, monitoring vaccine coverage, the fight against addictions, social determinants, etc.) could be entered in DMPs. Actually, as described above, the objective of DMPs is not clear enough to envisage this kind of use. However, its feasibility will be studied in the Rhône-Alpes region. Extraction of these types of data in regional popHR and linkage with data extracted from EHRs would consequently have to envisage the epidemiological platform as a population-based one, which would permit the study of more epidemiological and public health indicators than is actually possible.

Concerning the regional elaboration of databases issued from non-structured medical documents, at this stage, only experiments on samples have been conducted. The first step is to elaborate tools processing natural language data extracted from medical documents. The experiment is currently being performed in the context of the ALADIN-DTH project [20]. Preliminary results of this experiment showed feasibility. The tools developed for a specific topic (nosocomial infections) will be extended to other topics in coming years. A tool for de-identification of free-text medical documents has been developed and evaluated [51]. The feasibility of generalizing the data-mining techniques that we are using in the context of the ALADIN-DTH project will also be tested in the Regional Epidemiological Platform for other topics in coming months.

### Feasibility of setting up a regional epidemiological platform from a regional medical records management platform

Setting up shared EHRs is the object of major investment in many countries. However, implementation methods and deployment levels vary tremendously from one country to another. Australia (national HealthConnect program), New Zealand (national program), the United Kingdom (national program launched by the National Health Service), France (DMP project) and the United States have all launched projects in various forms, aimed at sharing EHRs among various care facilities to improve care coordination. In France, the DMP project has presented many difficulties that, to date, have prevented its development. Deployment of the regional SISRA platform has demonstrated that the process is feasible when designed and implemented on an appropriate geographic scale.

The use of HIS to meet epidemiological targets is undoubtedly more developed in the United States, Canada, Northern Europe and the United Kingdom. Epidemiological surveillance devices most often rely on the combination of various HISs. Each of these systems presents different methodological limitations and, when combined, can provide satisfactory indicators. In the United States, for example, a diabetes prevalence and incidence surveillance system is built on a mosaic of data sources, including data on mortality, hospitalization, diabetes-related disabilities, diabetes follow-up consultations and renal dialysis. The Centers for Disease Control and Prevention (CDC) have described the limitations of each information system [52], but combining these sources allows us to come sufficiently close to the disease's key epidemiological indicators for the purposes of developing and evaluating a public health policy. More recently, the CDC published an experiment regarding the automatic detection of certain mandatory-to-report diseases (chlamydia, gonorrhea, pelvic inflammatory disease, acute hepatitis A) in a group of 35 hospitals in Boston (Massachusetts) caring for 600,000 patients [53]. An external server was set up in various institutions to extract data on demographics, death, test prescriptions and results, diagnostic codes and types of medical care from each electronic record. Cases were defined by combining prescription information, bacteriological results and clinical data. When a case was detected, automated transmission was generated for reporting to the CDC. The same team published an experiment on the combination of biochemical data (AST, ALT, total bilirubin), serological data on hepatitis B (HbsAg, anti-Hbc) and clinical data from EHRs (icterus, no history of chronic hepatitis) for mandatory reporting of acute hepatitis B in the same group [54]. Various algorithms for automatic reporting were tested. Their sensitivity and specificity were compared with the traditional reporting system, and the results were very encouraging (sensitivity: 97.4%, 95% CI: 94-100%; specificity: 93.8%, 95% CI: 87-100%). More recently, Lau et al. [19] published an evaluation of EHR data from community oncology clinics against health claims data and cancer registry data. They showed that EHRs can provide detailed clinical data not found in other databases. These examples demonstrate the feasibility of this type of process and the potential for organizing such concerted development. However, these experiments were developed in a geographically-limited area or were not representative at the national level in the United States. Generalization of such experiments is subject to a number of barriers that need to be removed. A structured and coordinated strategy of the Division of Integrated Surveillance Systems and Services of the CDC's National Center for Public Health Informatics was created in 2005 [Internet site: http://www.cdc.gov/osels/phsipo/index.html] but the "fragmentation of population health data collection and data stewardship responsibilities among federal, state and local governments" stays one of the most important barriers to popHR in the United States [3].

### Encourage regional experiments in limited fields of application

Given the complexity and heterogeneity of hospital information systems as well as all the technical, political and ethical barriers to setting up a RPF-EPI, targets set for RPF-EHRs in epidemiology and public health must not be too ambitious and should be dealt with in successive stages. It seems important to start with limited experimental initiatives that allow all constraints to be considered in a very specific area. Attempting to consider every possible facet of introducing such a device would make implementation more complex and would paralyze experimental initiatives. Regional experiments should be encouraged before a more standardized national contour is designed. Several RPF-EHRs in France have the potential to meet this need.

### Encourage interdisciplinary research

RPF-EPI deployment requires interdisciplinary expertise that must be encouraged. In the United States, the National Research Council made the following recommendations in a recently-published report entitled *Computational Technology for Effective Health Care*: "encourage interdisciplinary research" notably in "the design of health care systems, processes and workflow"...encourage (or at least do not impede) efforts by healthcare organizations and communities to aggregate data about health care...processes and outcomes from all sources subject to appropriate protection of privacy and confidentiality" [55].

### Improve EHR computerization rates and quality

Medical record computerization rates are closely linked to care-system organization and government incentive policies. For example, New Zealand, Australia and the United Kingdom almost universally use EHRs in general medicine: 97%, 95% and 96% respectively [56]. This result, however, is linked to these countries' national funding programs designed to stimulate the adoption and application of EHRs. Professional medical organizations have also played a key role in rapid implementation. Furthermore, financial incentives based on the production of performance indicators from EHRs have been introduced [57]. A recent survey conducted in 11 countries estimated that 68% of French general practitioners use EHRs [56]. However, the level of electronic functionalities proposed in EHRs (alerts, prescription aid, decision-making help, patient selection by diagnosis, access to laboratory results, electronic prescription for additional exams, prevention-procedure reminders, etc.) was low in France. The country's level is average compared to others surveyed, where EHR usage rates range from 97% (Norway, New Zealand) to 37% (Canada). The United States comes in lower than France, at 46%. Although France's results from this study must be interpreted with caution, given the low participation rate of the physicians solicited, these numbers still put into perspective the very frequent discourse regarding France's delay in the area. Liberal French doctors have begun using EHRs; development can therefore be expected. However, major efforts must be made to deploy EHRs with a higher level of functionality to facilitate and improve the quality of data extraction, notably for epidemiological purposes. The new French National Convention of General Practitioners and Specialists [58] indirectly endorsed this approach, offering lump sum payments related to public health objectives. One public health objective is the organization of medical practice, particularly in terms of computerization. Article 12.4.1 of the Convention refers to medical records as a tool for coordination of care. Attending physicians must "establish a summary showing the medical treatment plan, including schedule tracking and interaction with other health professionals for advice and coordinated follow-up". It will be interesting in coming years to establish whether these incentive measures are effective for developing meaningful use of EHRs and to evaluate whether these data are practical for popHR.

Implementation of an epidemiological development system depends also on the EHR usage rate and the quality of data entered. Conversely, it is important to stress that, due to the validation processes required to enable data analysis, EHRs for epidemiological purposes may enhance the completeness and accuracy of data collected in electronic records [59]. In this sense, setting up a RPF-EPI will help improve the quality of patient care. There is thus no opposition between individual and collective EHR use, but rather complementary usage modes, both contributing to improved quality of the healthcare system.

### Oversee legal and ethical aspects associated with EHRs in public health

The collection, dissemination and any other processing of personal medical information must be carried out according to the rule of law, as set out in numerous legislative texts [60]. EHRs must be employed for public health purposes in accordance with the law. However, it should be noted that related security constraints are far more complex for EHR-sharing between healthcare professionals than for epidemiological use of the data. De-identification is the prerequisite for this type of use, and epidemiologists are familiar with such methods, in medico-administrative databases or registers, for example.

Furthermore, as pointed out by some French experts, the regional approach to EHR hosting offers the advantage of data-hosting network architecture, which helps limit losses or data piracy and hacker attacks [61].

## Summary

RPF-EHRs provide a good opportunity to meet certain epidemiological and public health needs. Whereas health professionals tend to dissociate individual patient care from population care, the RPF-EHR tool shows rather that they are complementary and mutually linked. It is by designing tools to process data intended for individual care that we will be able to assess and improve collective patient care systems. The regional level chosen for the creation of RPF-EHRs is coherent with regional strengthening of public health action, materialized through the creation of regional health agencies in France.

## Competing interests

The authors declare that they have no competing interests.

## Authors' contributions

MHM conceived the study and drafted the manuscript. TD participated in study conception and helped to draft the manuscript. SL and RS helped to draft the manuscript. PC participated in study conception and helped to draft the manuscript. All authors read and approved the final manuscript.

## Pre-publication history

The pre-publication history for this paper can be accessed here:

http://www.biomedcentral.com/1472-6947/12/28/prepub
